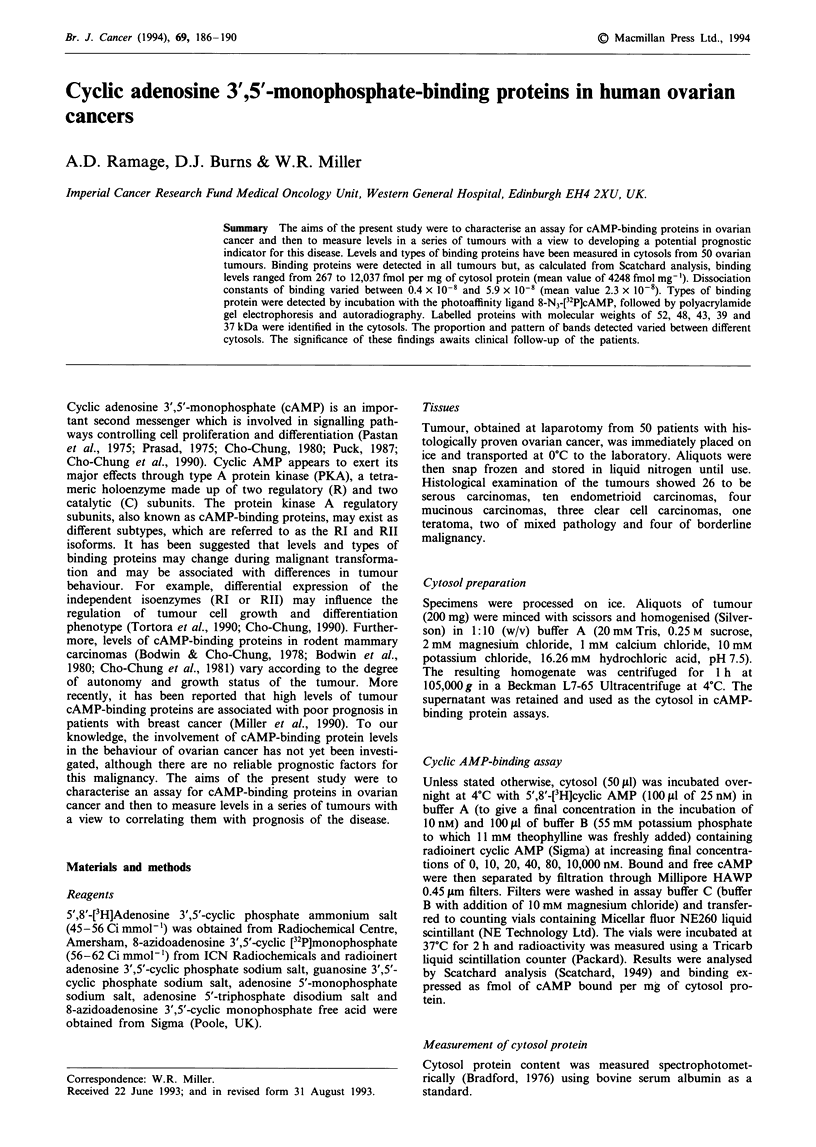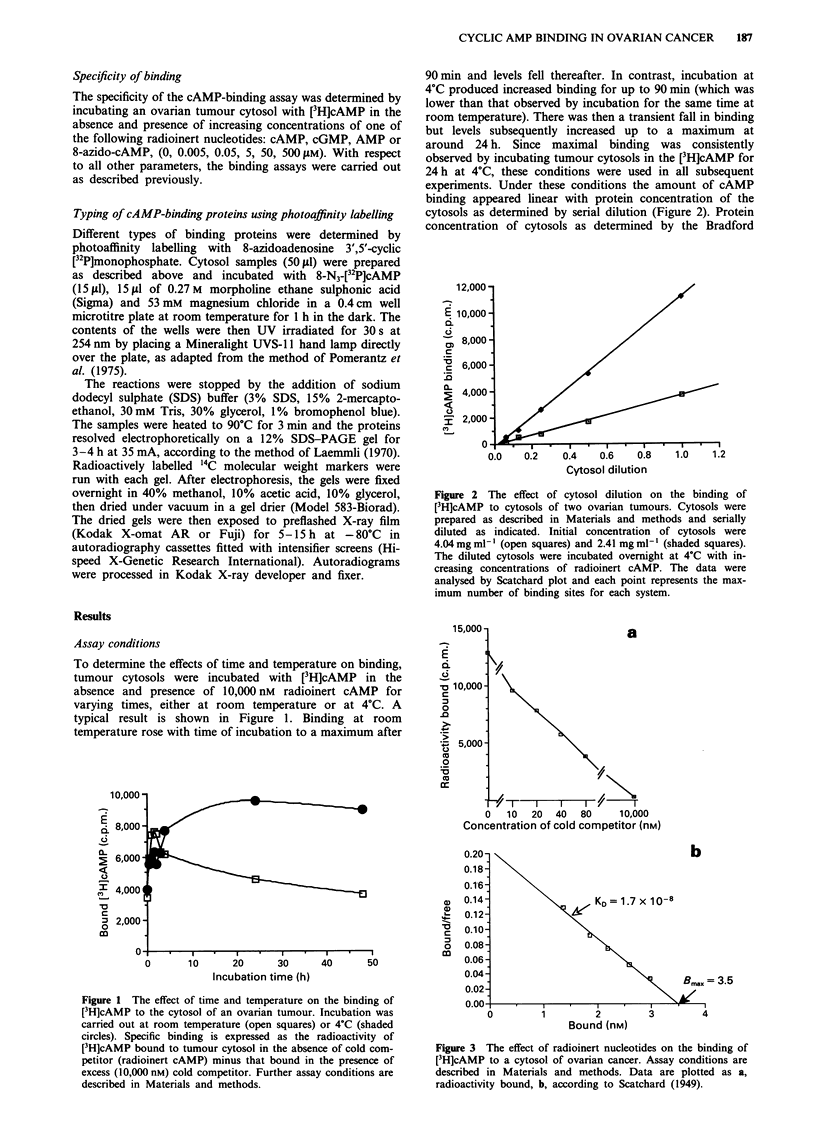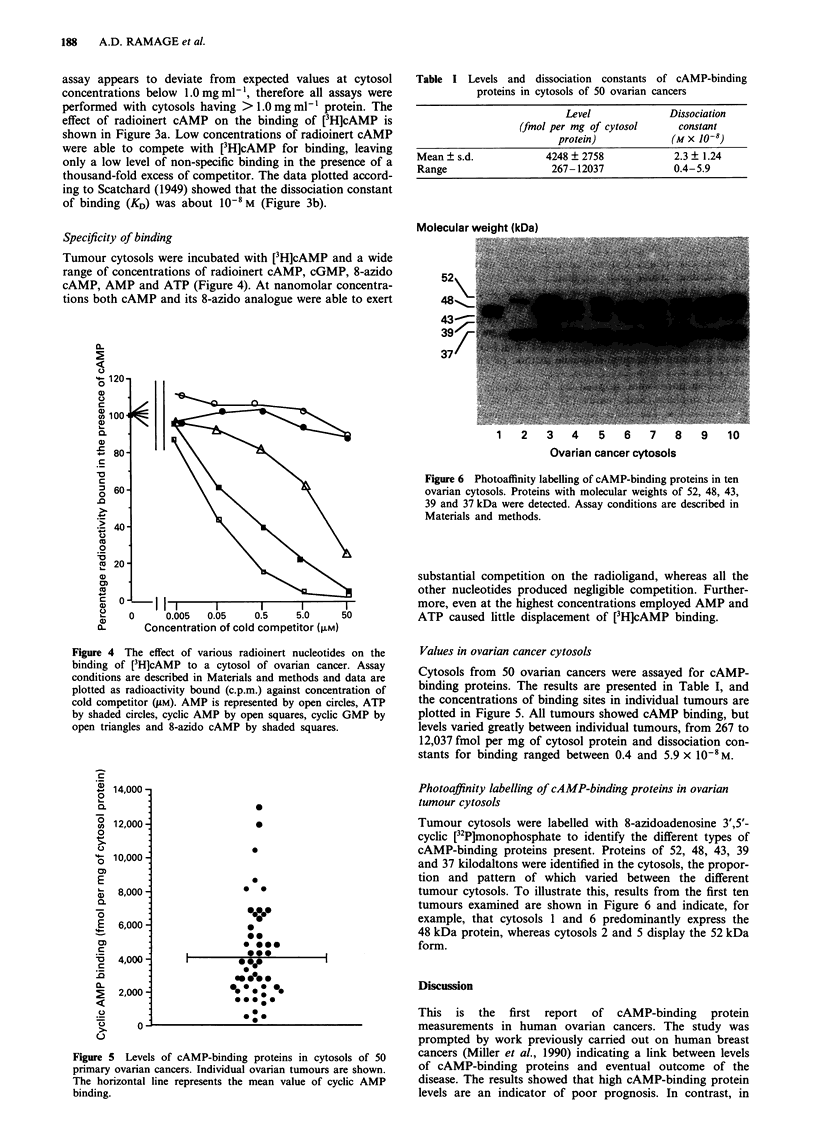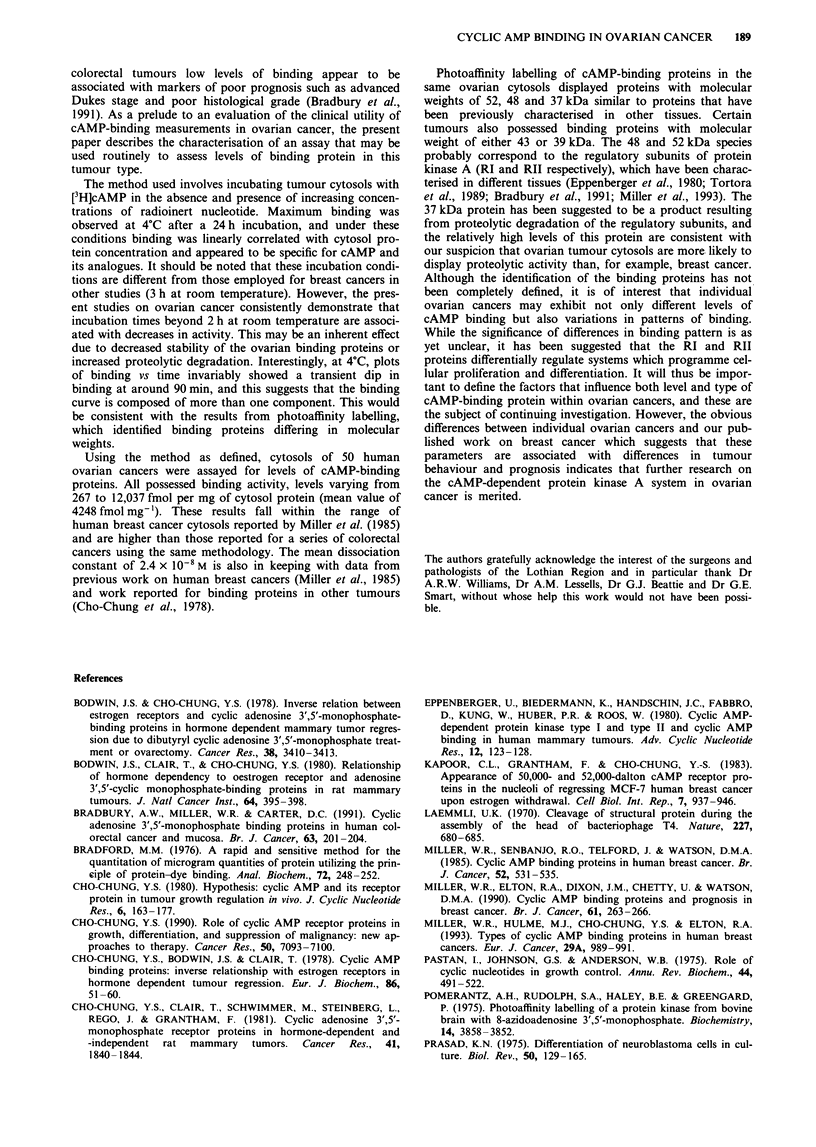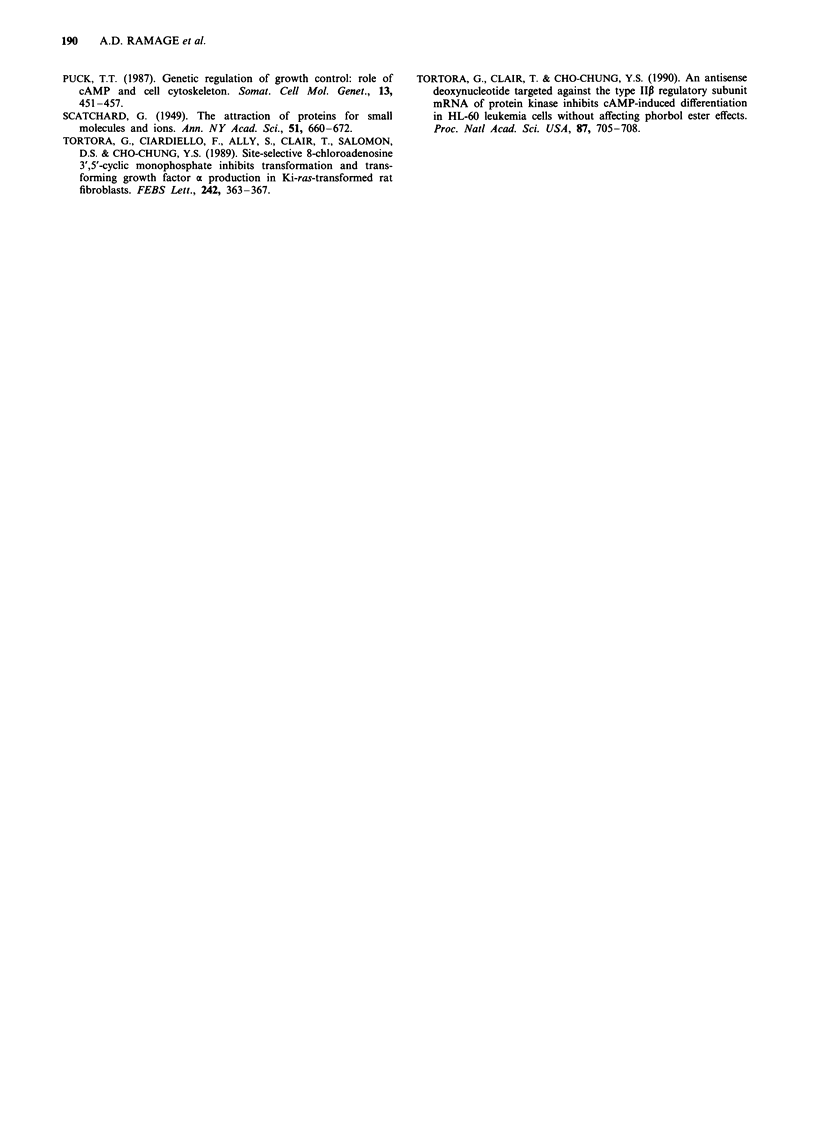# Cyclic adenosine 3',5'-monophosphate-binding proteins in human ovarian cancers.

**DOI:** 10.1038/bjc.1994.32

**Published:** 1994-01

**Authors:** A. D. Ramage, D. J. Burns, W. R. Miller

**Affiliations:** Imperial Cancer Research Fund Medical Oncology Unit, Western General Hospital, Edinburgh, UK.

## Abstract

**Images:**


Br. J. Cancer (1994), 69, 186–190 © Macmillan Press Ltd., 1994

### Cyclic adenosine 3′,5′-monophosphate-binding proteins in human ovarian cancers

A.D. Ramage, D.J. Burns & W.R. Miller

*Imperial Cancer Research Fund Medical Oncology Unit, Western General Hospital, Edinburgh EH4 2XU, UK.*

**Summary** The aims of the present study were to characterise an assay for cAMP-binding proteins in ovarian cancer and then to measure levels in a series of tumours with a view to developing a potential prognostic indicator for this disease. Levels and types of binding proteins have been measured in cytosols from 50 ovarian tumours. Binding proteins were detected in all tumours but, as calculated from Scatchard analysis, binding levels ranged from 267 to 12,037 fmol per mg of cytosol protein (mean value of 4248 fmol $mg^{-1}$). Dissociation constants of binding varied between $0.4 \times 10^{-8}$ and $5.9 \times 10^{-8}$ (mean value $2.3 \times 10^{-8}$). Types of binding protein were detected by incubation with the photoaffinity ligand 8-$N_3$-[$^{32}$P]cAMP, followed by polyacrylamide gel electrophoresis and autoradiography. Labelled proteins with molecular weights of 52, 48, 43, 39 and 37 kDa were identified in the cytosols. The proportion and pattern of bands detected varied between different cytosols. The significance of these findings awaits clinical follow-up of the patients.

Cyclic adenosine 3′,5′-monophosphate (cAMP) is an important second messenger which is involved in signalling pathways controlling cell proliferation and differentiation (Pastan *et al.*, 1975; Prasad, 1975; Cho-Chung, 1980; Puck, 1987; Cho-Chung *et al.*, 1990). Cyclic AMP appears to exert its major effects through type A protein kinase (PKA), a tetrameric holoenzyme made up of two regulatory (R) and two catalytic (C) subunits. The protein kinase A regulatory subunits, also known as cAMP-binding proteins, may exist as different subtypes, which are referred to as the RI and RII isoforms. It has been suggested that levels and types of binding proteins may change during malignant transformation and may be associated with differences in tumour behaviour. For example, differential expression of the independent isoenzymes (RI or RII) may influence the regulation of tumour cell growth and differentiation phenotype (Tortora *et al.*, 1990; Cho-Chung, 1990). Furthermore, levels of cAMP-binding proteins in rodent mammary carcinomas (Bodwin & Cho-Chung, 1978; Bodwin *et al.*, 1980; Cho-Chung *et al.*, 1981) vary according to the degree of autonomy and growth status of the tumour. More recently, it has been reported that high levels of tumour cAMP-binding proteins are associated with poor prognosis in patients with breast cancer (Miller *et al.*, 1990). To our knowledge, the involvement of cAMP-binding protein levels in the behaviour of ovarian cancer has not yet been investigated, although there are no reliable prognostic factors for this malignancy. The aims of the present study were to characterise an assay for cAMP-binding proteins in ovarian cancer and then to measure levels in a series of tumours with a view to correlating them with prognosis of the disease.

### Materials and methods

### *Reagents*

5′,8′-[$^3$H]Adenosine 3′,5′-cyclic phosphate ammonium salt (45–56 Ci $mmol^{-1}$) was obtained from Radiochemical Centre, Amersham, 8-azidoadenosine 3′,5′-cyclic [$^{32}$P]monophosphate (56–62 Ci $mmol^{-1}$) from ICN Radiochemicals and radioinert adenosine 3′,5′-cyclic phosphate sodium salt, guanosine 3′,5′-cyclic phosphate sodium salt, adenosine 5′-monophosphate sodium salt, adenosine 5′-triphosphate disodium salt and 8-azidoadenosine 3′,5′-cyclic monophosphate free acid were obtained from Sigma (Poole, UK).

Correspondence: W.R. Miller.
Received 22 June 1993; and in revised form 31 August 1993.

### *Tissues*

Tumour, obtained at laparotomy from 50 patients with histologically proven ovarian cancer, was immediately placed on ice and transported at 0°C to the laboratory. Aliquots were then snap frozen and stored in liquid nitrogen until use. Histological examination of the tumours showed 26 to be serous carcinomas, ten endometrioid carcinomas, four mucinous carcinomas, three clear cell carcinomas, one teratoma, two of mixed pathology and four of borderline malignancy.

### *Cytosol preparation*

Specimens were processed on ice. Aliquots of tumour (200 mg) were minced with scissors and homogenised (Silverson) in 1:10 (w/v) buffer A (20 mM Tris, 0.25 M sucrose, 2 mM magnesium chloride, 1 mM calcium chloride, 10 mM potassium chloride, 16.26 mM hydrochloric acid, pH 7.5). The resulting homogenate was centrifuged for 1 h at 105,000 *g* in a Beckman L7-65 Ultracentrifuge at 4°C. The supernatant was retained and used as the cytosol in cAMP-binding protein assays.

### *Cyclic AMP-binding assay*

Unless stated otherwise, cytosol (50 µl) was incubated overnight at 4°C with 5′,8′-[$^3$H]cyclic AMP (100 µl of 25 nM) in buffer A (to give a final concentration in the incubation of 10 nM) and 100 µl of buffer B (55 mM potassium phosphate to which 11 mM theophylline was freshly added) containing radioinert cyclic AMP (Sigma) at increasing final concentrations of 0, 10, 20, 40, 80, 10,000 nM. Bound and free cAMP were then separated by filtration through Millipore HAWP 0.45 µm filters. Filters were washed in assay buffer C (buffer B with addition of 10 mM magnesium chloride) and transferred to counting vials containing Micellar fluor NE260 liquid scintillant (NE Technology Ltd). The vials were incubated at 37°C for 2 h and radioactivity was measured using a Tricarb liquid scintillation counter (Packard). Results were analysed by Scatchard analysis (Scatchard, 1949) and binding expressed as fmol of cAMP bound per mg of cytosol protein.

### *Measurement of cytosol protein*

Cytosol protein content was measured spectrophotometrically (Bradford, 1976) using bovine serum albumin as a standard.

*Specificity of binding*

The specificity of the cAMP-binding assay was determined by incubating an ovarian tumour cytosol with [$^3$H]cAMP in the absence and presence of increasing concentrations of one of the following radioinert nucleotides: cAMP, cGMP, AMP or 8-azido-cAMP, (0, 0.005, 0.05, 5, 50, 500 μM). With respect to all other parameters, the binding assays were carried out as described previously.

*Typing of cAMP-binding proteins using photoaffinity labelling*

Different types of binding proteins were determined by photoaffinity labelling with 8-azidoadenosine 3',5'-cyclic [$^{32}$P]monophosphate. Cytosol samples (50 μl) were prepared as described above and incubated with 8-$N_3$-[$^{32}$P]cAMP (15 μl), 15 μl of 0.27 M morpholine ethane sulphonic acid (Sigma) and 53 mM magnesium chloride in a 0.4 cm well microtitre plate at room temperature for 1 h in the dark. The contents of the wells were then UV irradiated for 30 s at 254 nm by placing a Mineralight UVS-11 hand lamp directly over the plate, as adapted from the method of Pomerantz *et al.* (1975).

The reactions were stopped by the addition of sodium dodecyl sulphate (SDS) buffer (3% SDS, 15% 2-mercapto-ethanol, 30 mM Tris, 30% glycerol, 1% bromophenol blue). The samples were heated to 90°C for 3 min and the proteins resolved electrophoretically on a 12% SDS–PAGE gel for 3–4 h at 35 mA, according to the method of Laemmli (1970). Radioactively labelled $^{14}$C molecular weight markers were run with each gel. After electrophoresis, the gels were fixed overnight in 40% methanol, 10% acetic acid, 10% glycerol, then dried under vacuum in a gel drier (Model 583-Biorad). The dried gels were then exposed to preflashed X-ray film (Kodak X-omat AR or Fuji) for 5–15 h at −80°C in autoradiography cassettes fitted with intensifier screens (Hi-speed X-Genetic Research International). Autoradiograms were processed in Kodak X-ray developer and fixer.

### Results

*Assay conditions*

To determine the effects of time and temperature on binding, tumour cytosols were incubated with [$^3$H]cAMP in the absence and presence of 10,000 nM radioinert cAMP for varying times, either at room temperature or at 4°C. A typical result is shown in Figure 1. Binding at room temperature rose with time of incubation to a maximum after 90 min and levels fell thereafter. In contrast, incubation at 4°C produced increased binding for up to 90 min (which was lower than that observed by incubation for the same time at room temperature). There was then a transient fall in binding but levels subsequently increased up to a maximum at around 24 h. Since maximal binding was consistently observed by incubating tumour cytosols in the [$^3$H]cAMP for 24 h at 4°C, these conditions were used in all subsequent experiments. Under these conditions the amount of cAMP binding appeared linear with protein concentration of the cytosols as determined by serial dilution (Figure 2). Protein concentration of cytosols as determined by the Bradford

**Figure 1** The effect of time and temperature on the binding of [$^3$H]cAMP to the cytosol of an ovarian tumour. Incubation was carried out at room temperature (open squares) or 4°C (shaded circles). Specific binding is expressed as the radioactivity of [$^3$H]cAMP bound to tumour cytosol in the absence of cold competitor (radioinert cAMP) minus that bound in the presence of excess (10,000 nM) cold competitor. Further assay conditions are described in Materials and methods.

**Figure 2** The effect of cytosol dilution on the binding of [$^3$H]cAMP to cytosols of two ovarian tumours. Cytosols were prepared as described in Materials and methods and serially diluted as indicated. Initial concentration of cytosols were 4.04 mg ml$^{-1}$ (open squares) and 2.41 mg ml$^{-1}$ (shaded squares). The diluted cytosols were incubated overnight at 4°C with increasing concentrations of radioinert cAMP. The data were analysed by Scatchard plot and each point represents the maximum number of binding sites for each system.

**Figure 3** The effect of radioinert nucleotides on the binding of [$^3$H]cAMP to a cytosol of ovarian cancer. Assay conditions are described in Materials and methods. Data are plotted as **a**, radioactivity bound, **b**, according to Scatchard (1949).

assay appears to deviate from expected values at cytosol concentrations below 1.0 mg ml$^{-1}$, therefore all assays were performed with cytosols having >1.0 mg ml$^{-1}$ protein. The effect of radioinert cAMP on the binding of [$^3$H]cAMP is shown in Figure 3a. Low concentrations of radioinert cAMP were able to compete with [$^3$H]cAMP for binding, leaving only a low level of non-specific binding in the presence of a thousand-fold excess of competitor. The data plotted according to Scatchard (1949) showed that the dissociation constant of binding ($K_D$) was about $10^{-8}$ M (Figure 3b).

*Specificity of binding*

Tumour cytosols were incubated with [$^3$H]cAMP and a wide range of concentrations of radioinert cAMP, cGMP, 8-azido cAMP, AMP and ATP (Figure 4). At nanomolar concentrations both cAMP and its 8-azido analogue were able to exert

**Figure 4** The effect of various radioinert nucleotides on the binding of [$^3$H]cAMP to a cytosol of ovarian cancer. Assay conditions are described in Materials and methods and data are plotted as radioactivity bound (c.p.m.) against concentration of cold competitor (μM). AMP is represented by open circles, ATP by shaded circles, cyclic AMP by open squares, cyclic GMP by open triangles and 8-azido cAMP by shaded squares.

**Figure 5** Levels of cAMP-binding proteins in cytosols of 50 primary ovarian cancers. Individual ovarian tumours are shown. The horizontal line represents the mean value of cyclic AMP binding.

**Table I** Levels and dissociation constants of cAMP-binding proteins in cytosols of 50 ovarian cancers

| | *Level (fmol per mg of cytosol protein)* | *Dissociation constant (M × $10^{-8}$)* |
|---|---|---|
| Mean ± s.d. | 4248 ± 2758 | 2.3 ± 1.24 |
| Range | 267–12037 | 0.4–5.9 |

**Figure 6** Photoaffinity labelling of cAMP-binding proteins in ten ovarian cytosols. Proteins with molecular weights of 52, 48, 43, 39 and 37 kDa were detected. Assay conditions are described in Materials and methods.

substantial competition on the radioligand, whereas all the other nucleotides produced negligible competition. Furthermore, even at the highest concentrations employed AMP and ATP caused little displacement of [$^3$H]cAMP binding.

*Values in ovarian cancer cytosols*

Cytosols from 50 ovarian cancers were assayed for cAMP-binding proteins. The results are presented in Table I, and the concentrations of binding sites in individual tumours are plotted in Figure 5. All tumours showed cAMP binding, but levels varied greatly between individual tumours, from 267 to 12,037 fmol per mg of cytosol protein and dissociation constants for binding ranged between 0.4 and 5.9 × $10^{-8}$ M.

*Photoaffinity labelling of cAMP-binding proteins in ovarian tumour cytosols*

Tumour cytosols were labelled with 8-azidoadenosine 3′,5′-cyclic [$^{32}$P]monophosphate to identify the different types of cAMP-binding proteins present. Proteins of 52, 48, 43, 39 and 37 kilodaltons were identified in the cytosols, the proportion and pattern of which varied between the different tumour cytosols. To illustrate this, results from the first ten tumours examined are shown in Figure 6 and indicate, for example, that cytosols 1 and 6 predominantly express the 48 kDa protein, whereas cytosols 2 and 5 display the 52 kDa form.

### Discussion

This is the first report of cAMP-binding protein measurements in human ovarian cancers. The study was prompted by work previously carried out on human breast cancers (Miller et al., 1990) indicating a link between levels of cAMP-binding proteins and eventual outcome of the disease. The results showed that high cAMP-binding protein levels are an indicator of poor prognosis. In contrast, in colorectal tumours low levels of binding appear to be associated with markers of poor prognosis such as advanced Dukes stage and poor histological grade (Bradbury *et al.*, 1991). As a prelude to an evaluation of the clinical utility of cAMP-binding measurements in ovarian cancer, the present paper describes the characterisation of an assay that may be used routinely to assess levels of binding protein in this tumour type.

The method used involves incubating tumour cytosols with [$^3$H]cAMP in the absence and presence of increasing concentrations of radioinert nucleotide. Maximum binding was observed at 4°C after a 24 h incubation, and under these conditions binding was linearly correlated with cytosol protein concentration and appeared to be specific for cAMP and its analogues. It should be noted that these incubation conditions are different from those employed for breast cancers in other studies (3 h at room temperature). However, the present studies on ovarian cancer consistently demonstrate that incubation times beyond 2 h at room temperature are associated with decreases in activity. This may be an inherent effect due to decreased stability of the ovarian binding proteins or increased proteolytic degradation. Interestingly, at 4°C, plots of binding *vs* time invariably showed a transient dip in binding at around 90 min, and this suggests that the binding curve is composed of more than one component. This would be consistent with the results from photoaffinity labelling, which identified binding proteins differing in molecular weights.

Using the method as defined, cytosols of 50 human ovarian cancers were assayed for levels of cAMP-binding proteins. All possessed binding activity, levels varying from 267 to 12,037 fmol per mg of cytosol protein (mean value of 4248 fmol mg$^{-1}$). These results fall within the range of human breast cancer cytosols reported by Miller *et al.* (1985) and are higher than those reported for a series of colorectal cancers using the same methodology. The mean dissociation constant of $2.4 \times 10^{-8}$ M is also in keeping with data from previous work on human breast cancers (Miller *et al.*, 1985) and work reported for binding proteins in other tumours (Cho-Chung *et al.*, 1978).

Photoaffinity labelling of cAMP-binding proteins in the same ovarian cytosols displayed proteins with molecular weights of 52, 48 and 37 kDa similar to proteins that have been previously characterised in other tissues. Certain tumours also possessed binding proteins with molecular weight of either 43 or 39 kDa. The 48 and 52 kDa species probably correspond to the regulatory subunits of protein kinase A (RI and RII respectively), which have been characterised in different tissues (Eppenberger *et al.*, 1980; Tortora *et al.*, 1989; Bradbury *et al.*, 1991; Miller *et al.*, 1993). The 37 kDa protein has been suggested to be a product resulting from proteolytic degradation of the regulatory subunits, and the relatively high levels of this protein are consistent with our suspicion that ovarian tumour cytosols are more likely to display proteolytic activity than, for example, breast cancer. Although the identification of the binding proteins has not been completely defined, it is of interest that individual ovarian cancers may exhibit not only different levels of cAMP binding but also variations in patterns of binding. While the significance of differences in binding pattern is as yet unclear, it has been suggested that the RI and RII proteins differentially regulate systems which programme cellular proliferation and differentiation. It will thus be important to define the factors that influence both level and type of cAMP-binding protein within ovarian cancers, and these are the subject of continuing investigation. However, the obvious differences between individual ovarian cancers and our published work on breast cancer which suggests that these parameters are associated with differences in tumour behaviour and prognosis indicates that further research on the cAMP-dependent protein kinase A system in ovarian cancer is merited.

The authors gratefully acknowledge the interest of the surgeons and pathologists of the Lothian Region and in particular thank Dr A.R.W. Williams, Dr A.M. Lessells, Dr G.J. Beattie and Dr G.E. Smart, without whose help this work would not have been possible.

### References

BODWIN, J.S. & CHO-CHUNG, Y.S. (1978). Inverse relation between estrogen receptors and cyclic adenosine 3′,5′-monophosphate-binding proteins in hormone dependent mammary tumor regression due to dibutyryl cyclic adenosine 3′,5′-monophosphate treatment or ovarectomy. *Cancer Res.*, **38**, 3410–3413.

BODWIN, J.S., CLAIR, T., & CHO-CHUNG, Y.S. (1980). Relationship of hormone dependency to oestrogen receptor and adenosine 3′,5′-cyclic monophosphate-binding proteins in rat mammary tumours. *J. Natl Cancer Inst.*, **64**, 395–398.

BRADBURY, A.W., MILLER, W.R. & CARTER, D.C. (1991). Cyclic adenosine 3′,5′-monophosphate binding proteins in human colorectal cancer and mucosa. *Br. J. Cancer*, **63**, 201–204.

BRADFORD, M.M. (1976). A rapid and sensitive method for the quantitation of microgram quantities of protein utilizing the principle of protein-dye binding. *Anal. Biochem.*, **72**, 248–252.

CHO-CHUNG, Y.S. (1980). Hypothesis: cyclic AMP and its receptor protein in tumour growth regulation *in vivo*. *J. Cyclic Nucleotide Res.*, **6**, 163–177.

CHO-CHUNG, Y.S. (1990). Role of cyclic AMP receptor proteins in growth, differentiation, and suppression of malignancy: new approaches to therapy. *Cancer Res.*, **50**, 7093–7100.

CHO-CHUNG, Y.S., BODWIN, J.S. & CLAIR, T. (1978). Cyclic AMP binding proteins: inverse relationship with estrogen receptors in hormone dependent tumour regression. *Eur. J. Biochem.*, **86**, 51–60.

CHO-CHUNG, Y.S., CLAIR, T., SCHWIMMER, M., STEINBERG, L., REGO, J. & GRANTHAM, F. (1981). Cyclic adenosine 3′,5′-monophosphate receptor proteins in hormone-dependent and -independent rat mammary tumors. *Cancer Res.*, **41**, 1840–1844.

EPPENBERGER, U., BIEDERMANN, K., HANDSCHIN, J.C., FABBRO, D., KUNG, W., HUBER, P.R. & ROOS, W. (1980). Cyclic AMP-dependent protein kinase type I and type II and cyclic AMP binding in human mammary tumours. *Adv. Cyclic Nucleotide Res.*, **12**, 123–128.

KAPOOR, C.L., GRANTHAM, F. & CHO-CHUNG, Y.-S. (1983). Appearance of 50,000- and 52,000-dalton cAMP receptor proteins in the nucleoli of regressing MCF-7 human breast cancer upon estrogen withdrawal. *Cell Biol. Int. Rep.*, **7**, 937–946.

LAEMMLI, U.K. (1970). Cleavage of structural protein during the assembly of the head of bacteriophage T4. *Nature*, **227**, 680–685.

MILLER, W.R., SENBANJO, R.O., TELFORD, J. & WATSON, D.M.A. (1985). Cyclic AMP binding proteins in human breast cancer. *Br. J. Cancer*, **52**, 531–535.

MILLER, W.R., ELTON, R.A., DIXON, J.M., CHETTY, U. & WATSON, D.M.A. (1990). Cyclic AMP binding proteins and prognosis in breast cancer. *Br. J. Cancer*, **61**, 263–266.

MILLER, W.R., HULME, M.J., CHO-CHUNG, Y.S. & ELTON, R.A. (1993). Types of cyclic AMP binding proteins in human breast cancers. *Eur. J. Cancer*, **29A**, 989–991.

PASTAN, I., JOHNSON, G.S. & ANDERSON, W.B. (1975). Role of cyclic nucleotides in growth control. *Annu. Rev. Biochem.*, **44**, 491–522.

POMERANTZ, A.H., RUDOLPH, S.A., HALEY, B.E. & GREENGARD, P. (1975). Photoaffinity labelling of a protein kinase from bovine brain with 8-azidoadenosine 3′,5′-monophosphate. *Biochemistry*, **14**, 3858–3852.

PRASAD, K.N. (1975). Differentiation of neuroblastoma cells in culture. *Biol. Rev.*, **50**, 129–165.

PUCK, T.T. (1987). Genetic regulation of growth control: role of cAMP and cell cytoskeleton. *Somat. Cell Mol. Genet.*, **13**, 451–457.

SCATCHARD, G. (1949). The attraction of proteins for small molecules and ions. *Ann. NY Acad. Sci.*, **51**, 660–672.

TORTORA, G., CIARDIELLO, F., ALLY, S., CLAIR, T., SALOMON, D.S. & CHO-CHUNG, Y.S. (1989). Site-selective 8-chloroadenosine 3′,5′-cyclic monophosphate inhibits transformation and transforming growth factor α production in Ki-*ras*-transformed rat fibroblasts. *FEBS Lett.*, **242**, 363–367.

TORTORA, G., CLAIR, T. & CHO-CHUNG, Y.S. (1990). An antisense deoxynucleotide targeted against the type IIβ regulatory subunit mRNA of protein kinase inhibits cAMP-induced differentiation in HL-60 leukemia cells without affecting phorbol ester effects. *Proc. Natl Acad. Sci. USA*, **87**, 705–708.